# How to Disentangle Cation and Anion Dynamics of Fully Protonated Ionic Liquids: A Fast Field Cycling NMR Case Study

**DOI:** 10.1002/mrc.70072

**Published:** 2025-12-05

**Authors:** Lennart Kruse, Angel Mary Chiramel Tony, Daniel Rauber, Ralf Ludwig, Dietmar Paschek, Anne Strate

**Affiliations:** ^1^ Institut für Chemie, Physikalische und Theoretische Chemie Universität Rostock Rostock Germany; ^2^ Polymerchemie Universität des Saarlandes Saarbrücken Germany; ^3^ Saarene, Saarland Center for Energy Materials and Sustainability Universität des Saarlandes Saarbrücken Germany; ^4^ Department LL&M Universität Rostock Rostock Germany; ^5^ Leibniz Institut für Katalyse (LIKAT) Rostock Germany

**Keywords:** fast field cycling NMR relaxometry, hydrogen bonds, ionic liquids, molecular dynamics, rotation, translation

## Abstract

The molecular dynamics of ionic liquids (ILs) can be probed using fast field cycling (FFC) NMR relaxometry. Conventionally, such studies focus on ILs where only one ionic species carries NMR‐active nuclei or on systems combining 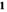
H nuclei on the cations with 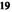
F nuclei on the anions. This way, the dynamics of cations and anions can be resolved individually. However, the situation becomes considerably more complex in fully protonated systems where both ions contain protons, because the various relaxation pathways can no longer be disentangled. Here we report the first FFC NMR investigation of such a case, using the IL triethylammonium methanesulfonate ([TEA][OMs]). Our strategy exploits selective partial deuteration of the ionic species, which enables the separate evaluation of cation and anion dynamics. We demonstrate for the first time that, from the known partial relaxation rates together with the determined interionic distances and self‐diffusion coefficients, the relaxation contribution arising from cation–anion interactions can be quantified. Remarkably, this approach even allows reconstruction of the total relaxation rate observed experimentally for the fully protonated IL. This methodology provides a fundamentally new route to overcoming the limited spectral resolution of FFC NMR relaxometry at low fields. More broadly, it establishes a framework for disentangling relaxation processes in complex multicomponent systems, thereby extending the applicability of FFC NMR to more challenging classes of ILs and related materials.

## Graphical abstract

This work presents the first FFC NMR relaxometry study of a fully protonated ionic liquid, [TEA][OMs]. Using selective partial deuteration, cation and anion dynamics were disentangled, enabling the hidden cation–anion relaxation contributions to be quantified and the total relaxation rate to be reconstructed.

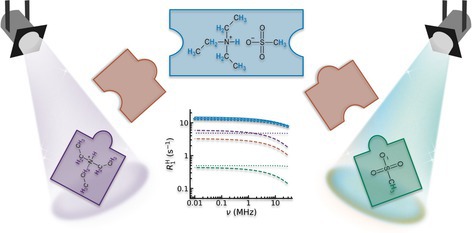

## Introduction

Ionic liquids (ILs) are salts composed solely out of ions with melting points typically below 100°C. Their unique physicochemical properties, such as negligible vapor pressure, good ionic conductivity, and broad chemical tunability [1, 2, 3, 4], have attracted sustained academic as well as industrial interest over the past decades. By chemically modifying either the cation or the anion, ILs can be tuned for applications ranging from catalysis and electrochemistry to advanced materials design [5, 6, 7].

At the molecular level, ILs are characterized by a subtle interplay of Coulombic interactions, dispersion forces, and hydrogen bonding. In particular, the introduction of functional groups or variations in alkyl chain length can significantly alter the strength and nature of these noncovalent interactions. Among them, hydrogen bonding has received growing attention [8, 9], as it strongly influences both microscopic ion–ion interactions and macroscopic bulk properties such as viscosity and density. Especially protic ILs, in which the cation carries an acidic proton, represent an important subclass where hydrogen bonding to the anion plays a central role. Using a variety of experimental and theoretical methods, the structures, strengths, and lifetimes of these hydrogen bonds can be investigated for different cations and anions [9, 10, 11, 12]. For example, in triethylammonium [TEA] 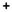
‐based ILs, the hydrogen bond strength between N–H and anion increases for different anions in the order of bis(trifluoromethylsulfonyl)imide ([NTf 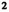] 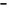) < trifluormethylsulfonate ([OTf] 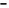) < methylsulfonate ([OMs] 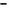). This trend directly correlates with enhanced viscosity and altered transport properties [13, 14].

Fast field cycling (FFC) NMR relaxometry is a powerful method to probe the molecular dynamics of ILs, as it allows the determination of both rotational correlation times and self‐diffusion coefficients from frequency‐dependent spin‐lattice relaxation data. However, the limited spectral resolution of FFC NMR relaxometry at low fields poses a real challenge. In many ILs, cation and anion dynamics can still be separated because only one ionic species contains protons, while the other often carries fluorine atoms. In such cases, 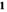
H and 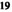
F relaxation measurements provide complementary information. This has been demonstrated for several different ILs in the past [15, 16, 17, 18, 19, 20, 21, 22, 23, 24, 25, 26, 27]. However, this approach is no longer possible for fluorine‐free ILs, such as [TEA][OMs], where both cation and anion contain protons. Here, the measured spin‐lattice relaxation rates provide an average of all the intramolecular and intermolecular contributions from protons, which makes it impossible to analyze ions specifically. Still, this IL is particularly interesting because of its strong hydrogen bonding ability and high viscosity.

**FIGURE 1:**
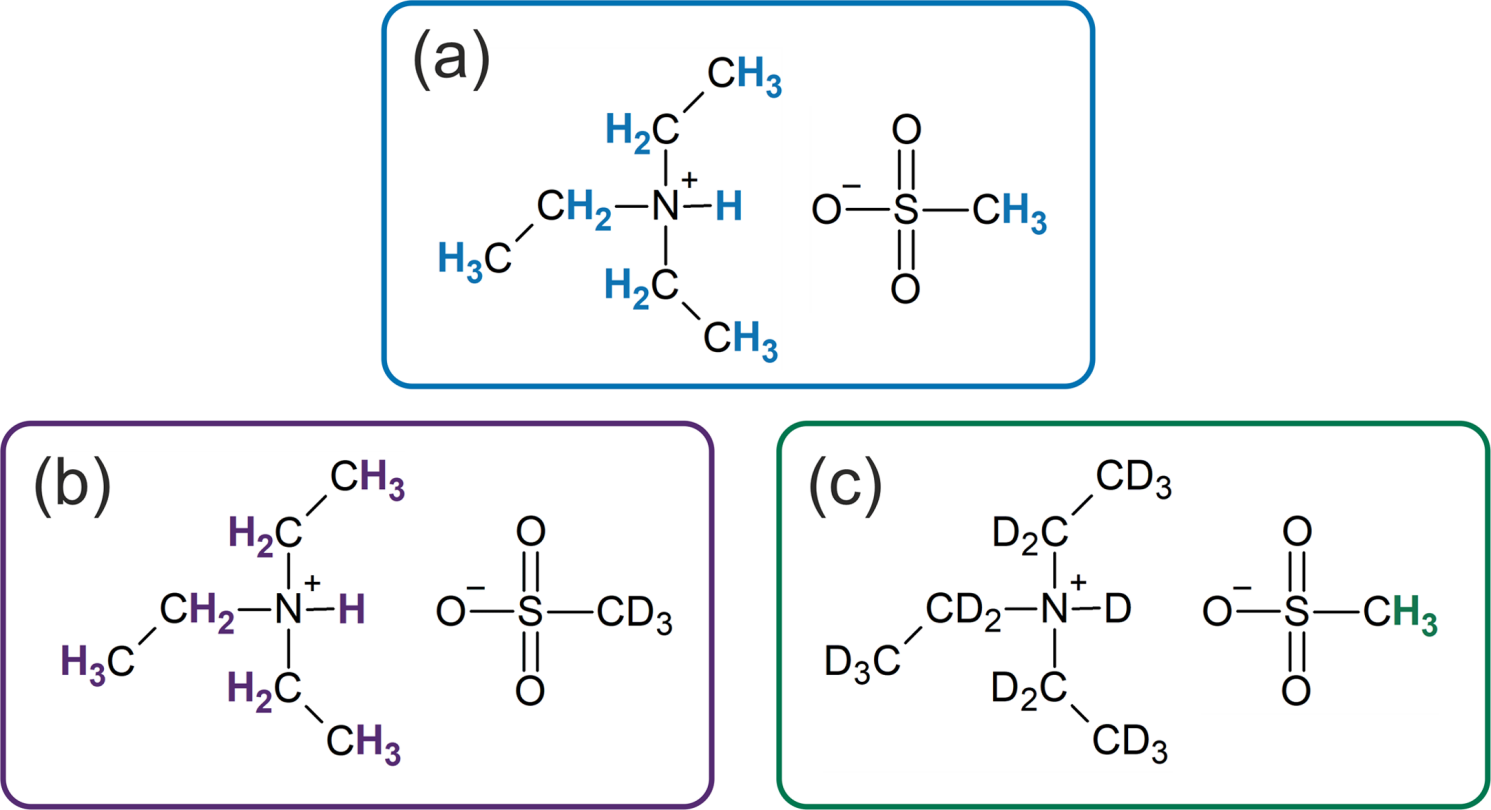
Molecular structure of the IL [TEA][OMs] being either (a) fully protonated on the cation and anion, (b) partially deuterated on the anion, or (c) partially deuterated on the cation.

In this work, we address this challenge by employing selective isotopic substitution through partial deuteration of either the cation or the anion. This strategy suppresses dipolar cation‐anion contributions and enables the extraction of ion‐specific relaxation dynamics. The chemical structures of all three samples are depicted in Figure 1.

In the IL with the deuterated anion ([TEA][OMs]‐ $d_{3}$), only the cation dynamics are probed, whereas in the IL with a deuterated cation ([TEA][OMs]‐ $d_{16}$), only the anion dynamics are accessible. Finally, by multiplying these ion‐specific relaxation contributions with appropriate weighting factors and quantifying the relaxation arising from intermolecular cation–anion interactions based on interionic distances and self‐diffusion coefficients of the individual species, we reconstruct the total relaxation rates of the fully protonated IL ([TEA][OMs]‐ $d_{0}$) based on the dynamical information we obtained from the partially deuterated systems.

## Relaxation Theory

In liquids, the spin‐lattice relaxation of 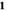
H nuclei at low magnetic fields is primarily governed by the dipole–dipole interactions between adjacent protons. These interactions are modulated by the translational and rotational motions of the proton‐bearing species. For a system, in which all of the protons are located on one specific molecular entity, the total frequency‐dependent spin‐lattice relaxation rate $R_{1}(\omega )$, which is the inverse of the spin‐lattice relaxation time $T_{1}(\omega )$, can be expressed as the sum of relaxation rates stemming from the intramolecular and intermolecular dipole–dipole contributions, corresponding to rotational and translational motions, respectively [28, 29, 30, 31].

(1) $$R_{1}^{\text{H}}(\omega )=\frac{1}{T_{1}^{\text{H}}(\omega )}=R_{1,\text{intra}}^{\text{H}}(\omega )+R_{1,\text{inter}}^{\text{H}}(\omega )$$

This description applies directly to the two partially deuterated ILs, [TEA][OMs]‐ $d_{3}$ and [TEA][OMs]‐ $d_{16}$, because in each case, the 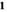
H nuclei are solely located either on the cation or the anion. For both intramolecular and intermolecular processes, the relaxation rate is given by a product of the dipolar coupling constant ( $A_{\mathrm{inter}/\mathrm{intra}}$) and a linear combination of spectral densities J, including the single and double quantum coherences.

(2) $$R_{1,\text{intra/inter}}^{\text{H}}(\omega )=A_{\mathrm{intra}/\mathrm{inter}}\times \left[J(\omega )+4J(2\omega )\right]$$

Here, the frequency‐independent constants $A_{\text{intra}}$ and $A_{\text{inter}}$ reflect structural information. For homonuclear 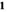
H– 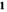
H interactions, they are defined as

(3) $$A_{\text{intra}}=\frac{3}{10}\frac{1}{r^{6}}{\left[\frac{\mu_{0}}{4\pi }\gamma_{\text{H}}^{2}\hbar \right]}^{2},$$

(4) $$A_{\text{inter}}=\frac{3}{10}\frac{N_{\text{H}}}{d^{3}}{\left[\frac{\mu_{0}}{4\pi }\gamma_{\text{H}}^{2}\hbar \right]}^{2},$$

where r is an effective intramolecular 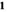
H– 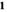
H distance, d is the intermolecular distance of closest approach, $N_{\text{H}}$ is the spin density of protons per unit volume, and $\gamma_{\text{H}}$ is the gyromagnetic ratio of protons. Any proton relaxation contributions originating from proton–deuteron couplings are negligible due to the much lower gyromagnetic ratio of deuterons ( $\gamma_{\text{D}}\approx 0.16\cdot \gamma_{\text{H}}$).

For modeling the translational and rotational molecular motions, certain spectral density functions are needed. Formally, the spectral density function J(ω) is the Fourier transform of the time‐dependent autocorrelation function of the fluctuating dipolar interaction. It is proportional to the probability of nuclear spin transitions occurring at the given frequency ω and quantifies how molecular motions modulate dipolar couplings and therefore the spin‐lattice relaxation rates.

Assuming isotropic molecular reorientation and rigid molecular geometry, the rotational contribution of both ions can be modeled by a Lorentzian, and the spectral density is given by

(5) $$J_{\text{intra}}=\frac{\tau_{\text{rot}}}{1+{\left(\omega \tau_{\text{rot}}\right)}^{2}},$$

where $\tau_{\text{rot}}$ is the rotational correlation time. Translational motion can be described within the force‐free hard‐sphere (FFHS) model developed by Hwang and Freed [32] and Ayant et al. [33], yielding the intermolecular spectral density

(6) $$J_{\text{inter}}^{\text{FFHS}}=72\overset{\infty }{\underset{0}{\int }}\frac{u^{2}}{81+9u^{2}-2u^{4}+u^{6}}\frac{u^{2}\tau_{\text{trans}}}{u^{4}+\omega^{2}\tau_{\text{trans}}^{2}}\text{d}u,$$

where $\tau_{\text{trans}}$ is the translational correlation time and u is an integration variable. The relative diffusion coefficient is related to $\tau_{\text{trans}}$ by

(7) $$D_{\text{rel}}=\frac{d^{2}}{\tau_{\text{trans}}},$$

with d being the distance of closest approach according to the FFHS model. For identical species (e.g., cation–cation or anion–anion), the relative diffusion coefficient is twice the self‐diffusion coefficient of the molecular species, $D_{\text{rel}}=2\cdot D_{\text{self}}$. Furthermore, translational self‐diffusion coefficients can also be extracted from the linear dependence of $R_{1}^{\text{H}}(\omega )$ on $\sqrt{\omega_{\text{H}}}$ [15, 30, 34, 35]:

(8) $$R_{1}^{\text{H}}(\omega )=R_{1}^{\text{H}}(0)-\frac{B_{\text{HH}}}{D_{\text{H}}^{\frac{3}{2}}}\cdot \sqrt{\omega }.$$

Here, $R_{1}^{\text{H}}(0)$ and $B_{\text{HH}}$ are frequency‐independent constants, where $B_{\text{HH}}$ is defined as

(9) $$B_{\text{HH}}=\left(\frac{1+4\sqrt{2}}{30}\right)\cdot {\left(\frac{\mu_{0}}{4\pi }\gamma_{\text{H}}^{2}\hbar \right)}^{2}\cdot \pi N_{\text{H}}.$$

For the fully protonated IL [TEA][OMs]‐ $d_{0}$, both ions contain 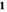
H nuclei, and only an averaged signal is observed due to the lack of spectral resolution at low fields. The measured magnetization is therefore a weighted average of cation and anion contributions. Strictly, a biexponential recovery would be expected. However, when the two $T_{1}$ values for cation and anion are similar, the data can be fitted monoexponentially, similar to the work of Jayakody et al. [23] and Leal Auccaise et al. [36], yielding only one single effective relaxation rate:

(10) $$R_{1,d0}^{\text{H}}(\omega )=W_{\text{c}}R_{1,\text{cation}}^{\text{H}}(\omega )+W_{\text{a}}R_{1,\text{anion}}^{\text{H}}(\omega ),$$

with weighting factors

(11) $$W_{\text{c}}=\frac{n_{\text{H}}^{\text{c}}}{n_{\text{H}}^{\text{c}}+n_{\text{H}}^{\text{a}}}=\frac{16}{19}\approx 0.84$$

(12) $$W_{\text{a}}=\frac{n_{\text{H}}^{\text{a}}}{n_{\text{H}}^{\text{c}}+n_{\text{H}}^{\text{a}}}=\frac{3}{19}\approx 0.16$$

where $n_{\text{H}}^{c}$ and $n_{\text{H}}^{a}$ are the number of protons on cation and anion, respectively. Finally, the total relaxation rate for the fully protonated IL must include not only cation–cation and anion–anion contributions but also cation–anion and anion–cation terms:

(13) $$\begin{array}{cc} R_{1,d0}^{\text{H}}(\omega ) & =W_{\text{c}}R_{1,\text{intra}}^{\text{cc}}(\omega )+W_{\text{c}}R_{1,\text{inter}}^{\text{cc}}(\omega )\\ & +W_{\text{c}}R_{1,\text{inter}}^{\text{ca}}(\omega )+W_{\text{a}}R_{1,\text{intra}}^{\text{aa}}(\omega )\\ & +W_{\text{a}}R_{1,\text{inter}}^{\text{aa}}(\omega )+W_{\text{a}}R_{1,\text{inter}}^{\text{ac}}(\omega ). \end{array}$$

Because of the large number of contributions and the missing spectral resolution, a direct fitting procedure for the fully protonated IL would not be feasible. Instead, we exploit for the first time the ion‐specific dynamics obtained from the partially deuterated systems to reconstruct the relaxation behavior of [TEA][OMs]‐ $d_{0}$.

## Methods

### Materials and Sample Preparation

**FIGURE 2:**
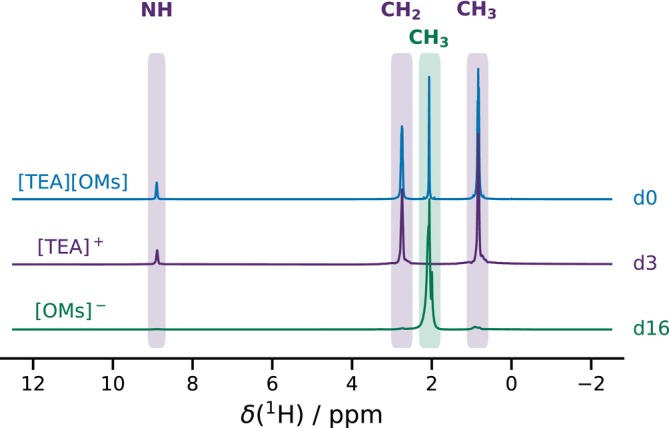
High‐field proton chemical shift spectra of [TEA][OMs]‐ $d_{0}$ (blue), [TEA][OMs]‐ $d_{3}$ (purple), and [TEA][OMs]‐ $d_{16}$ (green).

The IL triethylammonium methanesulfonate [TEA][OMs] was synthesized in fully protonated form ([TEA][OMs]‐ $d_{0}$) as well as in two selectively deuterated analogues, with either the anion methyl group deuterated ([TEA][OMs]‐ $d_{3}$) or the cation deuterated ([TEA][OMs]‐ $d_{16}$). Structures of all three ILs are shown in Figure 1. The syntheses followed established protocols described in the literature [15, 37]. In brief, protonated or deuterated precursors were used to ensure selective isotopic substitution on the target ionic species. Successful synthesis and selective deuteration were verified by high‐field 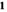
H NMR spectroscopy at a proton Larmor frequency of $\nu_{\text{H}}=500$ MHz. Figure 2 shows the obtained NMR spectra of the three samples. All four resonances are visible in the fully protonated IL, whereas the expected resonances are absent in the selectively deuterated species, confirming the isotopic substitution. Thus, in [TEA][OMs]‐ $d_{3}$, the observable protons are exclusively located on the cation, while in [TEA][OMs]‐ $d_{16}$, they are located exclusively on the anion.

To remove trace amounts of water and volatile impurities, all samples were dried under high vacuum ( $10^{-5}$ mbar) for 48 h. This procedure also minimizes dissolved oxygen, which can otherwise act as a paramagnetic relaxation source. After drying, the samples were immediately sealed off under vacuum in standard 10‐mm NMR tubes.

### FFC NMR Relaxometry Measurements

The frequency‐dependent 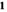
H spin‐lattice relaxation rates $R_{1}(\omega )$ were recorded using a Spinmaster FFC2000 (Stelar s.r.l., Mede, Italy) for all three ILs. One of these ILs ([TEA][OMs]‐ $d_{3}$) has been discussed previously in a different context [15]. In that earlier study, the focus was on determining self‐diffusion coefficients within the framework of the low‐frequency approach without dissecting total relaxation rates. Here, [TEA][OMs]‐ $d_{3}$ was considered as a representative example of purely proton‐bearing ILs.

In the present study, however, the focus is different, and for the first time, we reconstruct the relaxation behavior of a fully protonated IL from the ion‐specific contributions of its components. In this framework, the partially deuterated [TEA][OMs]‐ $d_{3}$ is one of two essential building blocks that enable the reconstruction. Therefore, the frequency‐dependent NMR dispersion curves of all ILs were obtained by measuring 30 data points, logarithmically distributed between 10 kHz and 38 MHz. Two experimental protocols were applied depending on the resonance frequency. Below 12 MHz, the magnetization was prepolarized at 25 MHz for a duration of at least $5\times T_{1}$, to ensure equilibration. At higher frequencies, a nonpolarized sequence was employed. In both cases, detection occurred at a fixed field of 16.3 MHz. Magnetization curves were constructed from 16 data points distributed linearly between $0.01\times T_{1}$ and $4\times T_{1}$. The relaxation curves were fitted monoexponentially, yielding the relaxation time $T_{1}$. For the fully protonated sample, biexponential recovery was in principle expected, but here also a monoexponential approximation was used (see the Supporting Information for representative fits). The uncertainty of individual $T_{1}$ values did not exceed 2%.

Temperature‐dependent measurements were performed from 293 to 343 K in 10 K steps. The temperature was regulated by a built‐in variable temperature control unit using heated compressed air. The actual sample temperature was verified using an external thermometer, and the accuracy was determined to be within 0.5 K. Measurements at lower temperatures than 293 K could not be achieved due to crystallization of the IL (melting point: 306 K [37]).

## Results and Discussion

### Ion‐Specific Dynamics in Partially Deuterated ILs

This study presents the first detailed analysis of cation and anion dynamics in a fully protonated IL using FFC NMR relaxometry. A key challenge arises from the inherently low spectral resolution of FFC NMR relaxometry. All protons contribute collectively to the total relaxation signal, regardless of whether they are located on the cation or the anion. To address this limitation, we synthesized two selectively deuterated ILs, [TEA][OMs]‐ $d_{3}$ and [TEA][OMs]‐ $d_{16}$, in which 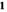
H nuclei are located either on the cation or on the anion, respectively. This isotopic substitution suppresses cation–anion contributions between different species and allows for a separate quantification of ion‐specific dynamics.

**FIGURE 3:**
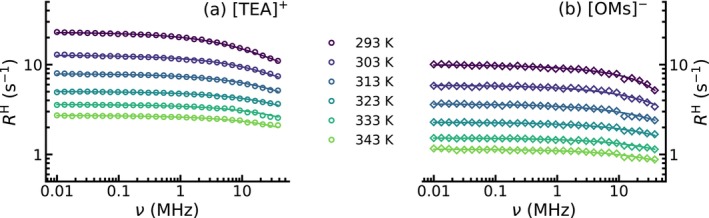
Temperature‐dependent NMRD profiles for 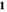
H nuclei of (a) the cation in [TEA][OMs]‐ $d_{3}$ plotted as circles and (b) the anion in [TEA][OMs]‐ $d_{16}$ plotted as diamonds. Solid lines represent global fits according to Equations ((1), (2), (3), (4), (5), (6)).

**FIGURE 4:**
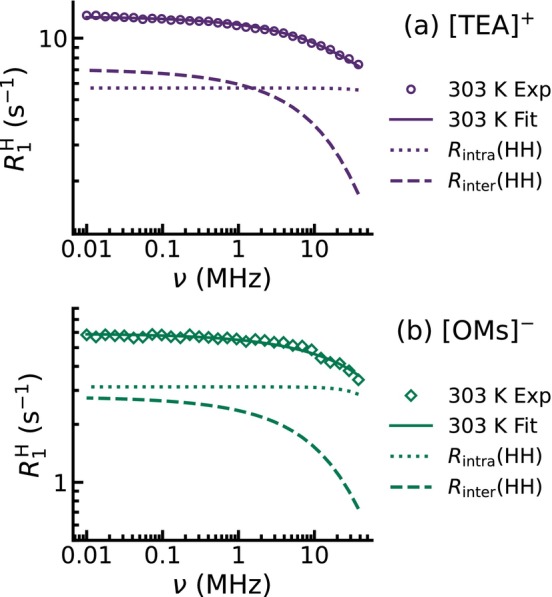
Dissected 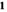
H NMRD profiles at 303 K for (a) [TEA][OMs]‐ $d_{3}$ and (b) [TEA][OMs]‐ $d_{16}$. Experimental data for (a) the cation and (b) the anion are plotted as open symbols, while the total fits are shown as solid lines. The contributions from homonuclear intramolecular relaxation $R_{\text{intra}}$ are shown as dotted lines, and homonuclear intermolecular contributions $R_{\text{inter}}$ are represented as dashed lines.

The frequency‐dependent proton spin‐lattice relaxation rates $R_{1}(\omega )$ were measured for both partially deuterated ILs over the temperature range between 293 and 343 K. Corresponding NMR dispersion profiles are shown in Figure 3a for the [TEA] 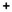 cation and in Figure 3b for the [OMs] 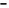 anion. The data were analyzed and fitted according to Equations ((1), (2), (3), (4), (5), (6)). During the fitting procedure, data for all temperatures were fitted simultaneously using the temperature‐independent structural parameters ( r and d) and the dynamical correlation times $\tau_{\text{rot}}$ and $\tau_{\text{trans}}$ as the fitting parameters. The final values for all of them can be found in Section S2 of the Supporting Information. Additionally, for each IL, the temperature‐dependent spin densities $N_{\text{H}}$ were derived from the experimentally obtained macroscopic densities (see Table S1 in the Supporting Information). Clearly, for the [TEA] 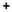 cation ( $d_{3}$), the overall relaxation rates are substantially larger than for the [OMs] 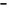 anion ( $d_{16}$), reflecting more efficient dipolar relaxation pathways. For both systems, the NMRD profiles also show pronounced temperature dependence. At lower temperatures, dispersion becomes more prominent, consistent with the well‐established slowing down of molecular motions in viscous molecular and ionic liquids. According to Equation (1), the total spin‐lattice relaxation rates were dissected into two different homonuclear relaxation contributions arising from intramolecular and intermolecular relaxation pathways. For the larger [TEA] 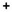 cation, the intermolecular relaxation dominates at low frequencies and exhibits strong dispersion (Figure 4a). In contrast, the intramolecular contribution remains to a great extent frequency‐independent and dominates the relaxation behavior at higher frequencies. The situation is completely different for the [OMs] 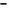 anion (Figure 4b). Here, all of the protons are only located in one single methyl group. Consequently, the intramolecular contribution dominates over the whole frequency range, because intermolecular interactions are statistically less pronounced. Once again, the intermolecular relaxation rate shows stronger dispersion. The total and dissected spin‐lattice relaxation rates are shown for the cation and for the anion for all temperatures in Figures S3 and S4 in the Supporting Information.

**FIGURE 5:**
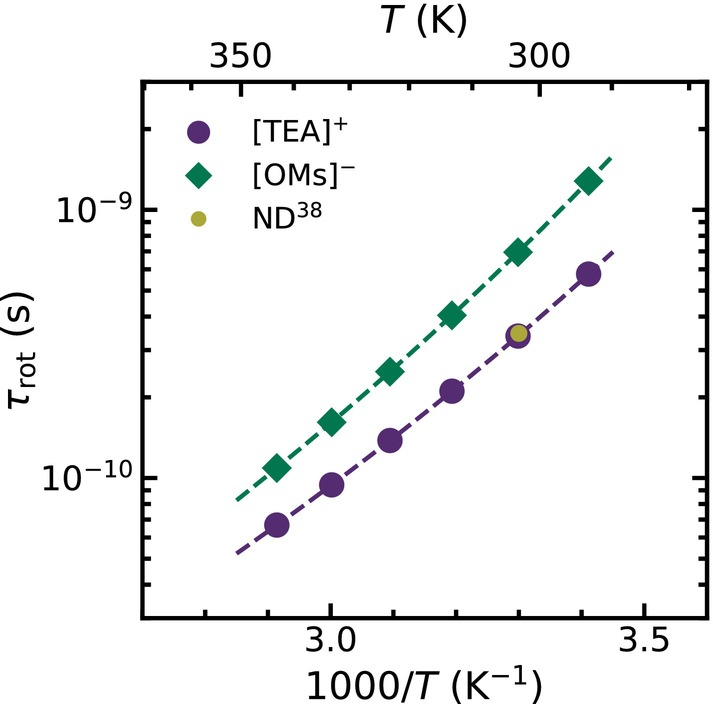
Temperature‐dependent rotational correlation times for the [TEA] 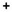 cation (purple dots) in [TEA][OMs]‐ $d_{3}$ and for the [OMs] 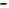 anion (green diamonds) in [TEA][OMs]‐ $d_{16}$. Both data sets follow a VFT temperature behavior (dashed line). The data match the reorientational correlation time obtained from deuteron quadrupole relaxation time measurements (yellow). [38].

A precise analysis of the intramolecular relaxation rates according to Equation (5) allows for a detailed description of the rotational dynamics of the different ions. The obtained overall rotational correlation times $\tau_{\text{rot}}$ for both of them are presented in Figure 5. They clearly show a Vogel–Fulcher–Tammann (VFT) temperature behavior. Corresponding fitting parameters can be found in Table S3 in the Supporting Information. Remarkably, the larger [TEA] 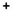 cation shows faster rotational dynamics compared with the smaller [OMs] 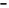 anion. Additionally, we added a data point for the deuteron reorientational correlation time of the N–D bond vector in the [TEA] 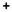 cation obtained from NMR deuteron quadrupole relaxation time measurements at 303 K [38]. This correlation time nicely overlaps with the rotational correlation times derived by FFC NMR realxometry. The almost perfect agreement supports the idea that both describe the same overall isotropic rotational dynamics of the whole cation.

**FIGURE 6:**
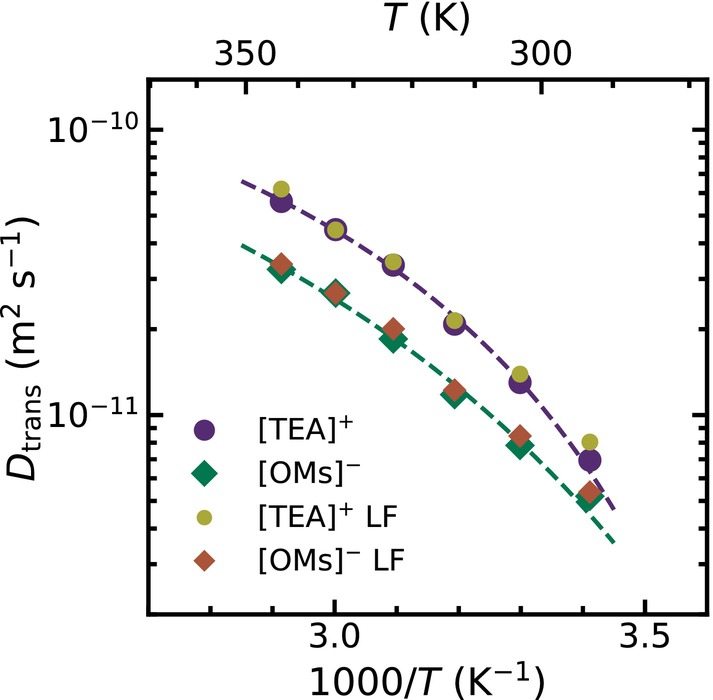
Temperature‐dependent self‐diffusion coefficients determined for the [TEA] 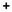 cation (purple dots) in [TEA][OMs]‐ $d_{3}$ and for the [OMs] 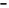 anion (green diamonds) in [TEA][OMs]‐ $d_{16}$. Both data sets follow a VFT temperature behavior (dashed line). Self‐diffusion coefficients were calculated following Equations (6) and (7) and from the low‐frequency slopes according to Equation (8) (LF).

In Figure 6, we show the translational self‐diffusion coefficients $D_{\text{H}}$ for the [TEA] 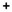 cation and for the [OMs] 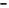 anion. Diffusion slows down for lower temperatures and can again be described by a VFT temperature behavior (see Table S3 in the Supporting Information). In line with the rotational dynamics, the cation diffuses faster than the anion. An additional way to obtain self‐diffusion coefficients, especially when protons are involved, is using pulsed field gradient methods. In our case, however, the agreement between self‐diffusion coefficients extracted by dissecting the total relaxation rates and those obtained from the low‐frequency approach according to Equation (8) strengthens confidence in the quantitative reliability of the analysis.

### Reconstructing the Total Relaxation Rate of Fully Protonated [TEA][OMs]

Based on the ion‐specific dynamics obtained from the deuterated systems, we finally reconstruct the total relaxation behavior of the fully protonated IL [TEA][OMs]‐ $d_{0}$. According to Equation (13), the overall relaxation rate is a weighted sum of six different contributions: intramolecular and intermolecular terms for both cation–cation ( $R_{1,\text{intra}}^{\text{cc}},R_{1,\text{inter}}^{\text{cc}}$) and anion–anion ( $R_{1,\text{intra}}^{\text{aa}},R_{1,\text{inter}}^{\text{aa}}$) interaction, plus two cross terms describing cation–anion relaxation. Four of these terms were directly determined from the partially deuterated systems, while the cation‐anion relaxation contributions can now be estimated from structural and dynamical considerations. Specifically, the cation–anion distance of closest approach $d_{\text{ca}}$ was approximated as the geometric mean $d_{\text{ca}}=\sqrt{d_{\text{cc}}\cdot d_{\text{aa}}}$ of the previously determined cation–cation ( $d_{\text{cc}}$) and anion–anion ( $d_{\text{aa}}$) distances. The relative diffusion coefficient $D_{\text{rel}}=D_{\text{c}}+D_{\text{a}}$ was taken as the sum of the independently determined self‐diffusion coefficients of cation and anion. From these parameters, the translational correlation time can be derived according to the established relation

(14) $$\tau_{\text{trans}}^{\text{ca}}=\tau_{\text{trans}}^{\text{ac}}=\frac{d_{\text{ca}}^{2}}{D_{\text{c}}+D_{\text{a}}}.$$

**FIGURE 7:**
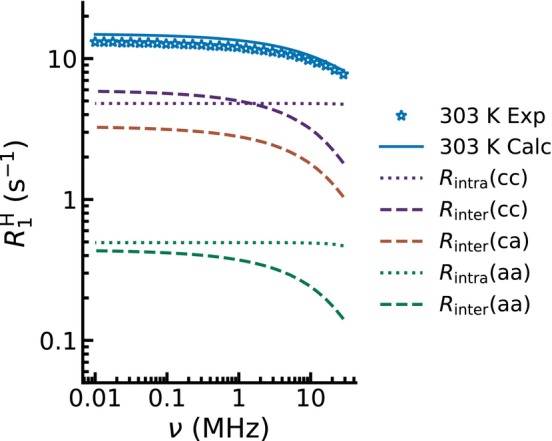
Comparison of experimentally measured relaxation rates of fully protonated [TEA][OMs] (blue stars) and reconstructed relaxation rates (blue, solid line) calculated from the dynamics obtained for the partially deuterated ILs, including the weighted partial relaxation contributions stemming from cation (violet) and anion (green) considering the intermolecular (dashed) and intramolecular (dotted) interactions. The calculated interaction between cation and anion is shown in orange.

Assuming that the individual contributions ( $R_{1,\text{inter}}^{\text{ca}}$ and $R_{1,\text{inter}}^{\text{ac}}$) scale with the fraction of 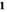
H nuclei involved, the reconstructed overall relaxation rate shows excellent agreement with experimental data for fully protonated [TEA][OMs] across the entire frequency range and temperature series. For 303 K, this reconstruction is presented in Figure 7. Here, the partial relaxation rates derived from [TEA][OMs]‐ $d_{3}$ are shown in violet, those from [TEA][OMs]‐ $d_{16}$ in green, while the newly calculated contribution based on the above approximations is displayed in orange. This latter contribution includes $R_{1,\text{inter}}^{\text{ca}}$ and $R_{1,\text{inter}}^{\text{ac}}$ in one single term. The sum of all contributions is shown as a solid blue line and shows remarkable agreement with the experimentally determined values for the fully protonated IL [TEA][OMs], which are indicated by blue stars. The corresponding plots for all the other investigated temperatures are provided in Figure S5 in the Supporting Information. Our procedure is highly effective across the entire temperature range.

## Conclusions

In this work, we have demonstrated a strategy to disentangle cation and anion dynamics in the fully protonated IL [TEA][OMs] using FFC NMR relaxometry in combination with selective isotopic substitution. By synthesizing partially deuterated ions, we were able to separately probe the dynamical behavior of the [TEA] 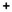 cation and the [OMs] 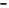 anion. This approach provided ion‐specific rotational correlation times $\tau_{\text{rot}}$ and self‐diffusion coefficients $D_{\text{trans}}$, both of which exhibit VFT temperature dependence. Consistently, the cation displays faster rotational and translational dynamics compared with the anion.

Importantly, we show that the partial relaxation contributions from the partially deuterated systems can be used to reconstruct the total relaxation rates of the fully protonated IL with remarkable accuracy. This reconstruction required explicit consideration of cation–anion relaxation rates, which were estimated firstly from intermolecular cation–anion distances based on the geometric mean of cation–cation and anion–anion distances and secondly relative diffusion coefficients based on cation and anion self‐diffusion coefficients. Finally, the total relaxation rates are treated as the weighted sum of partial relaxation rates from the cation and anion. The agreement between reconstructed and experimentally measured relaxation dispersions confirms the validity of this approach and highlights the power of combining FFC NMR relaxometry with deuteration experiments.

Beyond the specific case of [TEA][OMs], our findings establish a framework for probing dynamics in multicomponent systems where different species carry protons as NMR‐active nuclei. This represents a significant step toward extending FFC relaxometry to classes of ILs and related materials where conventional dissection of the NMRD profiles is not feasible.

## Author Contributions

**Lennart Kruse:** data curation FFC (supporting), formal analysis (lead), investigation (lead), visualization (supporting), writing – original draft (equal), writing – review and editing (supporting). **Angel Mary Chiramel Tony:** data curation FFC (lead). **Daniel Rauber:** IL synthesis (lead). **Ralf Ludwig:** conceptualization (supporting), supervision (equal), resources (lead), writing – review and editing (supporting). **Dietmar Paschek:** conceptualization (supporting), project administration (supporting), supervision (supporting), writing – review and editing (supporting). **Anne Strate:**conceptualization (lead), funding acquisition (lead), project administration (lead), visualization (lead), software (lead), supervision (equal), writing – original draft (equal), writing – review and editing (lead).

## Conflicts of Interest

The authors declare no conflicts of interest.

## Supporting information



MRC70072‐sup‐0001‐supporting_information.pdf

## Data Availability

Measured spin‐lattice relaxation data are available upon request from the authors. Data supporting this article have been included as part of the Supporting Information: density information, rotational correlation times, self‐diffusion coefficients, magnetization curves, dissected relaxation rates and reconstructed relaxation rates.
