# Supplementary material for: How to Disentangle Cation and Anion Dynamics of Fully Protonated Ionic Liquids: A Fast Field Cycling NMR Case Study
Source: Magn Reson Chem. 2025 Dec 5;64(3):265–72. doi: 10.1002/mrc.70072 (PMC12867595; doi:10.1002/mrc.70072)
Supplement: Supplementary file 1 — MRC70072‐sup‐0001‐supporting_information.pdf [file MRC-64-265-s001.pdf]

Supporting Information

How to disentangle cation and anion dynamics of fully protonated ionic liquids: A fast field cycling NMR case study

Lennart Kruse,[a] Angel Mary Chiramel Tony,[a] Daniel Rauber,[bc]
Dietmar Paschek,[a] Ralf Ludwig[ade] and Anne Strate*[a]

[a]*Institut für Chemie, Physikalische und Theoretische Chemie, Universität Rostock, Albert-Einstein-Straße 27, D-18059 Rostock, Germany*

[b]*Polymerchemie, Universität des Saarlandes, Campus C4 2, D-66123 Saarbrücken, Germany*

[c]*Saarene, Saarland Center for Energy Materials and Sustainability, Universität des Saarlandes, D-66123 Saarbrücken, Germany*

[d]*Department LL&M, Universität Rostock, Albert-Einstein-Straße 25, D-18059 Rostock, Germany*

[e]*Leibniz Institut für Katalyse (LIKAT), Albert-Einstein-Straße 29a, D-18059 Rostock, Germany*

*Corresponding author:
Anne Strate, Email: anne.strate@uni-rostock.de

Contents

S1 Density information 2

S2 Rotational correlation times and self-diffusion coefficients 2

S3 Magnetization curves 3

S4 Dissected spin-lattice relaxation rates of partially deuterated [TEA][OMs]-d_3 and [TEA][OMs]-d_{16} 5

S5 Reconstruction of the total relaxation rates of fully protonated [TEA][OMs] 7

S1 Density information

For modeling and fitting of the relaxation rates for the intermolecular interactions, the spin density N needs to be considered. The spin density is defined as

$$N_\mathrm{H} = \frac{\rho \cdot N_\mathrm{A} \cdot n_\mathrm{H}}{M}, \tag{1}$$

where ρ is the macroscopic density of the ionic liquid, N_A is Avogadro's constant, n_H is the number of protons per species and M is the molecular mass of one ion pair. The densities ρ have been measured for the fully protonated IL previously[1] and have been included in Tab. S1 for overview. A linear fit has been applied. For the calculation of the spin densities it was assumed, that the density does not change upon deuteration. However, the molecular mass of the ion pair as well as the number of protons per ion pair does change. The temperature-dependent spin densities may be found in Tab. S1.

Table S1: Measured and fitted macroscopic densities and calculated spin densities of protons (N_H) in protonated and partially deuterated [TEA][OMs].

	$\rho_\mathrm{exp}\,/\,\mathrm{g\,cm^{-3}}$		$N_\mathrm{H}\,/\,10^{28}\,\mathrm{m^{-3}}$		
$T\,/\,\mathrm{K}$	Exp.	Fit	[TEA][OMs] - d0	[TEA][OMs] - d3	[TEA][OMs] - d16
293	1.1211	1.1209	6.5010	5.3920	0.9490
298	1.1177	-	-	-	-
303	1.1143	1.1143	6.4626	5.3602	0.9434
308	1.1109	-	-	-	-
313	1.1076	1.1077	6.4243	5.3284	0.9378
318	1.1043	-	-	-	-
323	1.1010	1.1011	6.3859	5.2965	0.9322
328	1.0977	-	-	-	-
333	1.0945	1.0945	6.3475	5.2647	0.9266
338	1.0912	-	-	-	-
343	1.0880	1.0879	6.3091	5.2329	0.9210

S2 Rotational correlation times and self-diffusion coefficients

The procedure for fitting the measured NMR dispersion profiles has been described previously[2] and yields temperature-dependent rotational correlation times as well as self-diffusion coefficients. In addition, the two structural parameters r and d are obtained. While r is an effective intramolecular coupling distance, d is the distance of closest approach between two intermolecularly coupled species. It is assumed that both parameters are temperature-independent, and the obtained values are listed in Tab. S2.

Table S2: Fitted structural parameters in the two deuterated ionic liquids [TEA][OMs]-d3 and [TEA][OMs]-d16.

IL	[TEA][OMs]-d3	[TEA][OMs]-d16
$r\,/\,10^{-10}\,\mathrm{m}$	1.92	2.40
$d\,/\,10^{-10}\,\mathrm{m}$	4.64	3.48

The obtained rotational correlation times τ_rot and self-diffusion coefficients D_trans are fitted following a Vogel-Fulcher-Tamann (VFT) temperature behavior according to eqs. (2) and (3).

The obtained VFT fitting parameters are listed in Tab. S3.

$$\tau_{\text{rot}} = \tau_{\text{rot},0} \cdot \exp\left(\frac{B}{T - T_0}\right) \tag{2}$$

$$D_{\text{trans}} = D_{\text{trans},0} \cdot \exp\left(-\frac{B}{T - T_0}\right) \tag{3}$$

Table S3: VFT fitting parameters for rotational correlation times τ_{rot} and translational self-diffusion coefficients D_{trans} for cations in [TEA][OMs]-d3 and anions in [TEA][OMs]-d16 obtained from FFC NMR dispersion profiles.

Ion	$\tau_{\text{rot},0}/\,\text{s}$	$B/\,\text{K}$	$T_0/\,\text{K}$
$[\text{TEA}]^+$	6.7263E-14	1449.77	133.05
$[\text{OMs}]^-$	4.9204E-14	1589.43	136.81

Ion	$D_{\text{trans},0}/\,\text{m}^2\text{s}^{-1}$	$B/\,\text{K}$	$T_0/\,\text{K}$
$[\text{TEA}]^+$	5.0651E-10	219.80	242.98
$[\text{OMs}]^-$	7.0010E-10	385.81	216.80

S3 Magnetization curves

The magnetization curves are shown for fully protonated [TEA][OMs] at three different temperatures (Figs. S1 and S2). For a better comparison, the magnetization values are normalized to the maximum value.

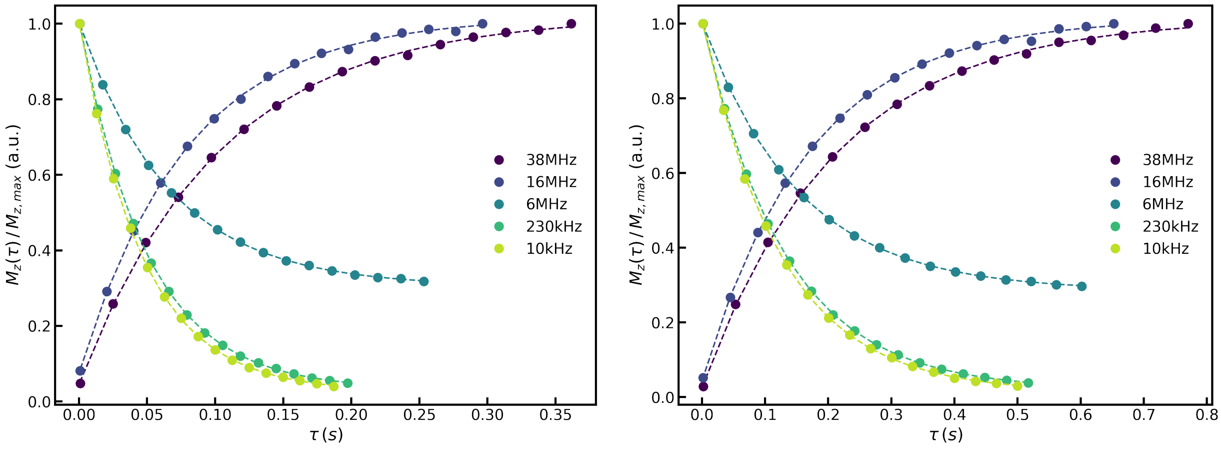

Figure S1: Normalized magnetization curves at corresponding Proton Larmor frequencies at 293 K (left) and 313 K (right).

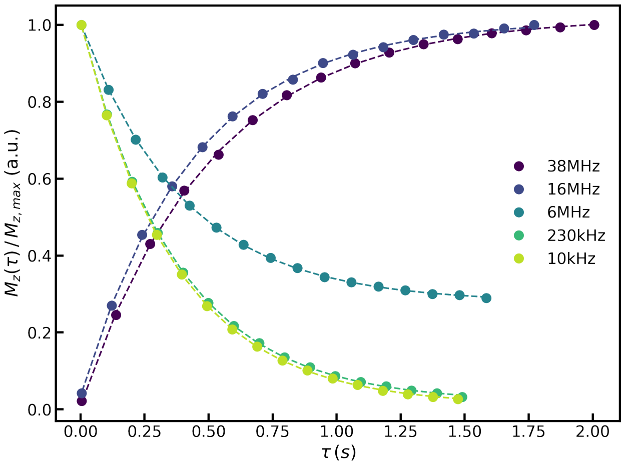

Figure S2: Normalized magnetization curves at corresponding Proton Larmor frequencies at 343 K.

The curves are plotted against the delay time τ at five different frequencies. At low frequencies the magnetization relaxes due to the use of a prepolarized pulse sequence, while at high frequencies the magnetization builds up. In all cases, the curves were fitted with a monoexponential function. Obtained T_1-times with corresponding relative estimation errors can be found in Tab. S4. The error remained below 2 % for all temperatures and frequencies.

Table S4: T_1-times including fitting/estimation errors for the IL [TEA][OMs].

	293 K		313 K		343 K	
ν_H / MHz	T_1 / ms	rel. err T_1 / %	T_1 / ms	rel. err T_1 / %	T_1 / ms	rel. err T_1 / %
38.01	102.25	1.20	210.75	1.33	506.77	0.80
16.20	78.75	1.39	168.32	1.37	435.70	0.91
6.91	65.84	0.58	152.38	0.52	400.59	0.59
0.23	49.65	0.38	130.82	0.29	372.30	0.39
0.01	46.03	0.50	124.74	0.60	367.25	0.37

S4 Dissected spin-lattice relaxation rates of partially deuterated [TEA][OMs]-d_3 and [TEA][OMs]-d_{16}

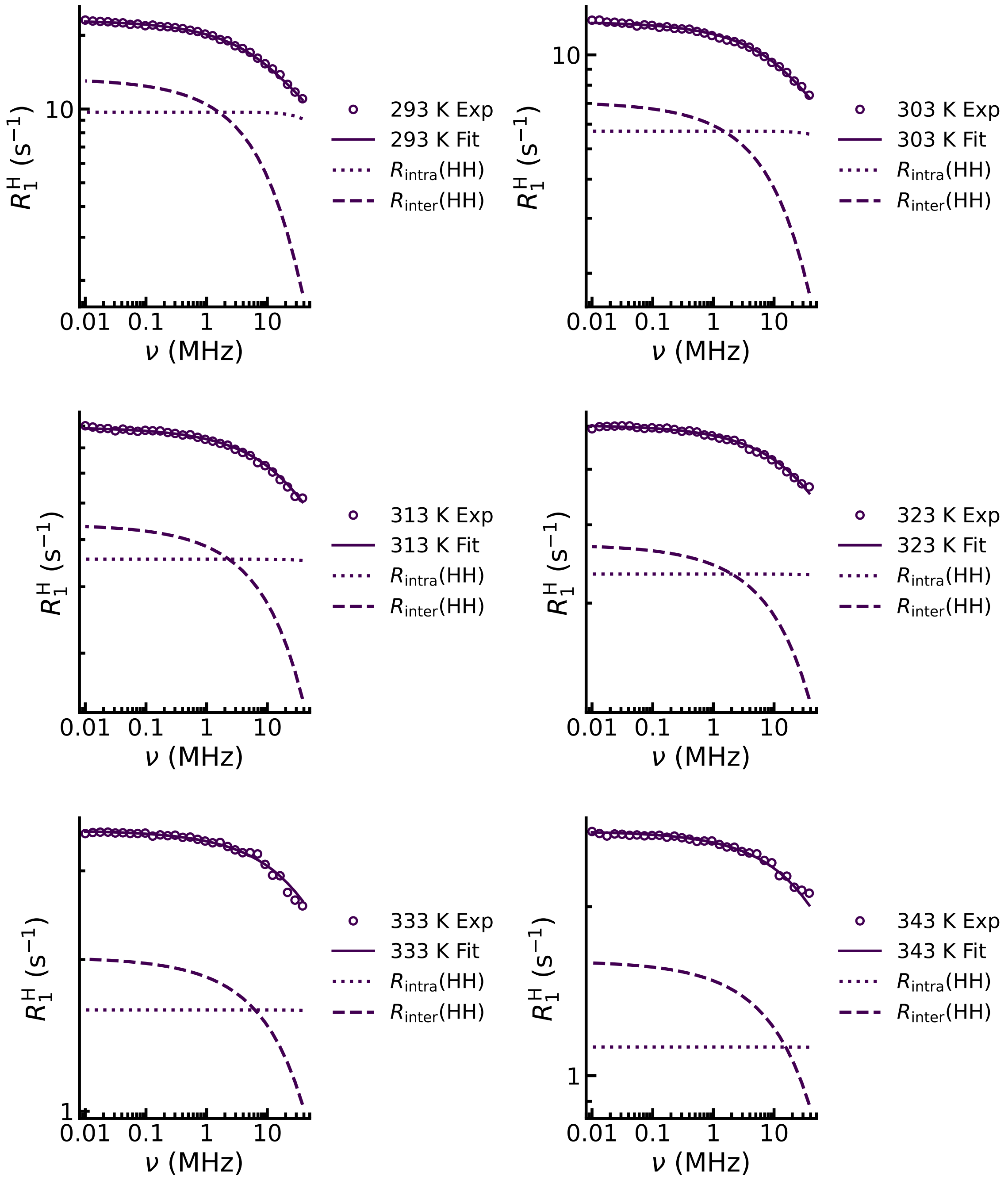

Figure S3: Frequency-dependent dissected relaxation rates for ^{1}H nuclei of the cation in [TEA][OMs]-d3. Each subplot corresponds to a different temperature. Experimental data are plotted as open circles, while the total fits corresponding to Eqs. (1)-(6) are shown as purple solid lines. The contributions from homonuclear intramolecular relaxation R_{intra} are shown as dotted lines and homonuclear intermolecular contributions R_{inter} are represented by dashed lines.

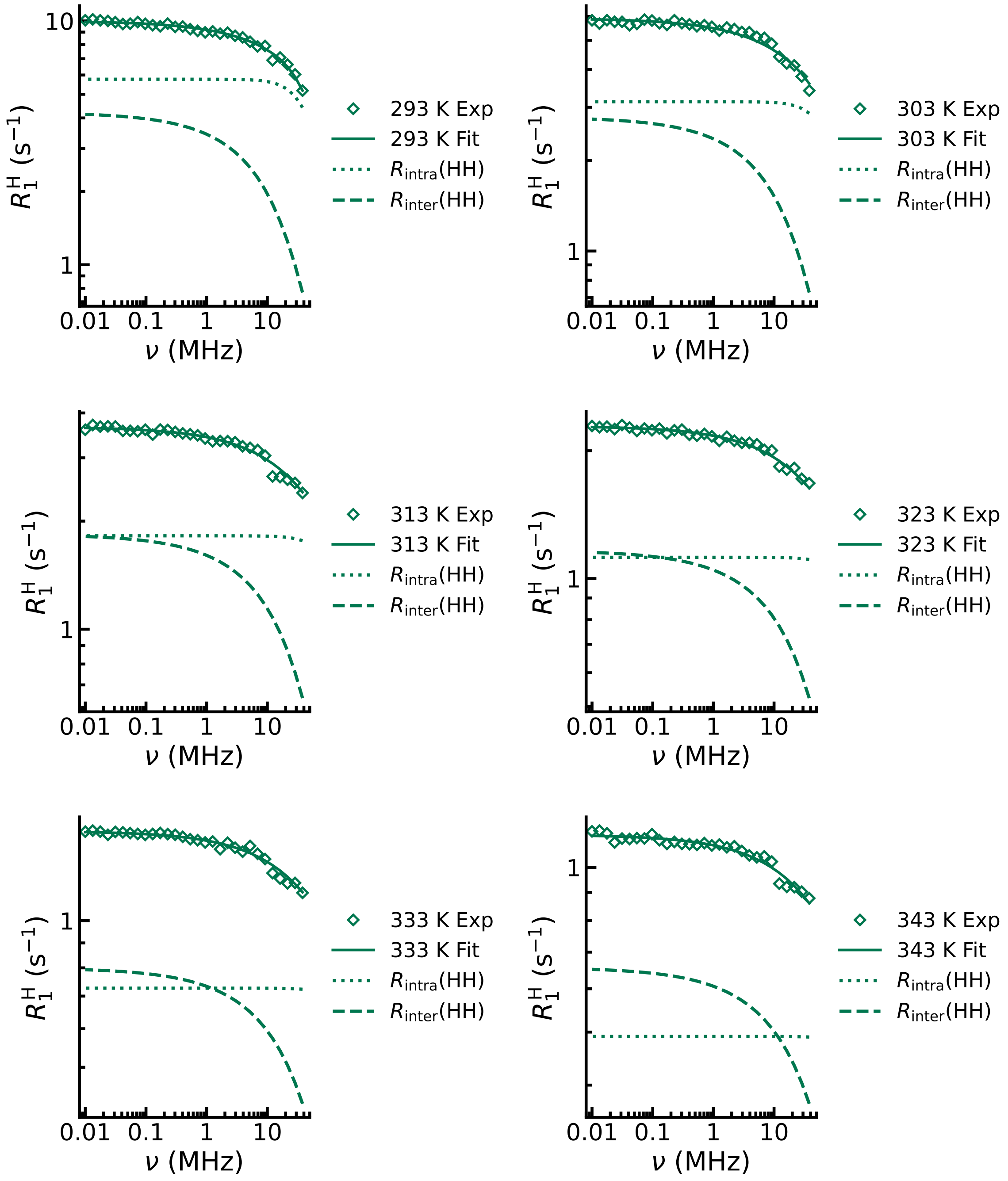

Figure S4: Frequency-dependent dissected relaxation rates for ^{1}H nuclei of the anion in [TEA][OMs]-d16. Each subplot corresponds to a different temperature. Experimental data are plotted as open diamonds, while the total fits corresponding to Eqs. (1)-(6) are shown as green solid lines. The contributions from homonuclear intramolecular relaxation R_{intra} are shown as dotted lines, and homonuclear intermolecular contributions R_{inter} are represented by dashed lines.

S5 Reconstruction of the total relaxation rates of fully protonated [TEA][OMs]

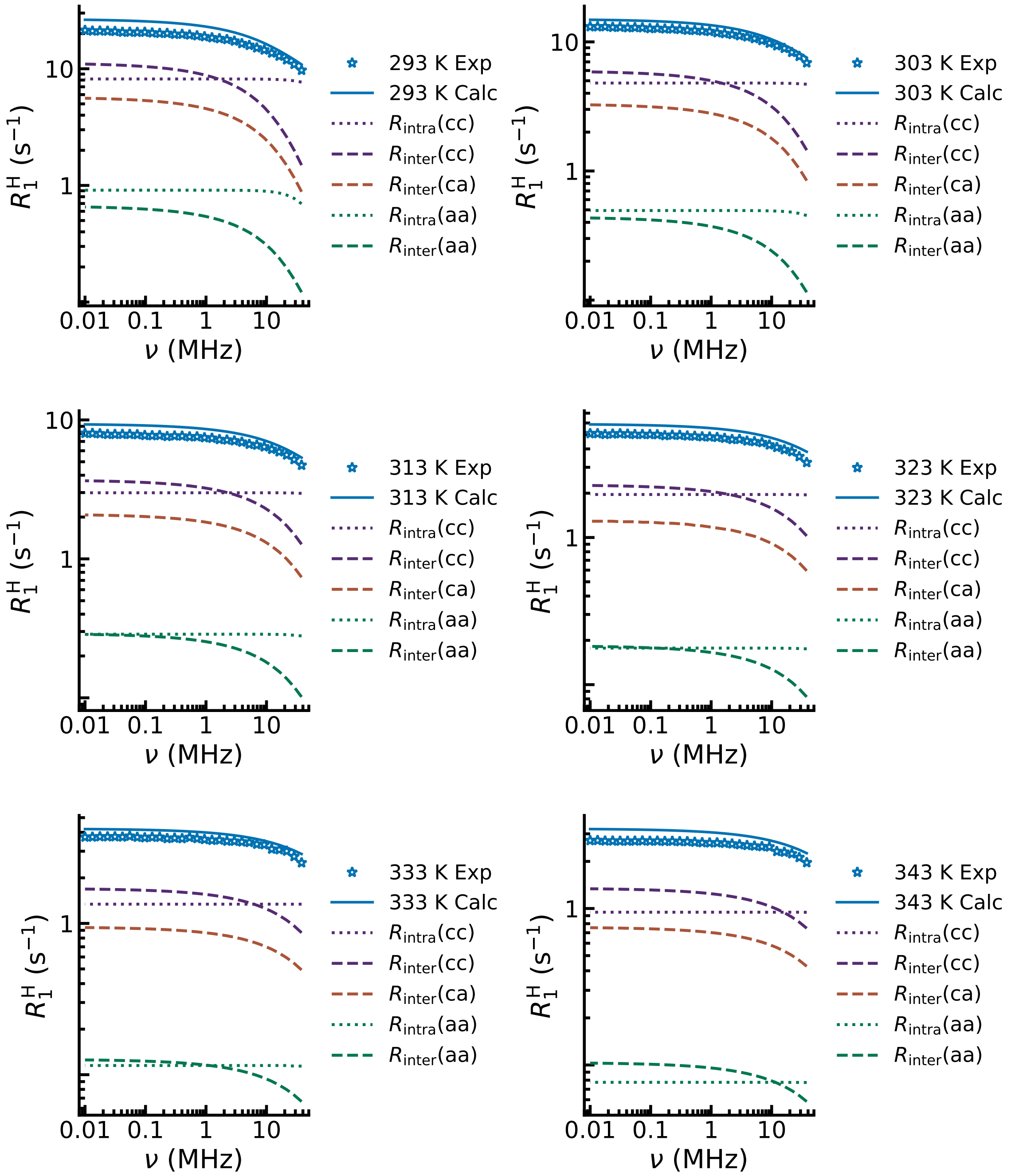

Figure S5: Reconstructed relaxation rates (blue, dashed line) in comparison with experimentally measured relaxation rates of fully protonated [TEA][OMs] (blue stars) at all temperatures. This includes the weighted partial relaxation contributions stemming from the cation (violet) and anion (green), considering the inter- (dashed) and intramolecular (dotted) interactions. The calculated interaction between cation and anion is shown in orange.

References

[1] L. Kruse, A. M. Chiramel Tony, D. Paschek, P. Stange, R. Ludwig, A. Strate, *Journal of Physical Chemistry Letters* **2024**, *15*, 10410.

[2] L. Kruse, T. van Alphen, J. Busch, D. Paschek, R. Ludwig, A. Strate, *Physical Chemistry Chemical Physics* **2025**, *27*, 10927.
